# On the Influence of Additives and Modifiers on the Chiral HPLC Separation of the Enantiomers of Nicotine

**DOI:** 10.1002/chir.70020

**Published:** 2025-01-26

**Authors:** Mehdi Ashraf‐Khorassini, William M. Coleman, Weston J. Umstead

**Affiliations:** ^1^ Department of Chemistry Virginia Tech Blacksburg Virginia USA; ^2^ iiiconsulting, LLC Conway South Carolina USA; ^3^ Daicel Chiral Technologies West Chester Pennsylvania USA

**Keywords:** chiral, enantiomer, high‐performance liquid chromatography, nicotine, separation

## Abstract

The influence of additives and modifiers on the chiral HPLC separation of the nicotine enantiomers using UV/Vis detection is discussed. Selected alcohols as modifiers and selected amines as additives were found to have a significant effect on the resolution and retention times of nicotine enantiomers even to the point of eliminating component elution. Systematic variations in the concentration of ethanol, methanol, and isopropanol, as modifiers, along with variations in the concentration of diethylamine, triethylamine, tributylamine, ethylenediamine, isopropylamine, as additives, revealed that the average resolution (*R*) of the nicotine enantiomers ranged from 2.9 to 7.57, using a mobile phase flow rate of 0.80 mL/min. The average retention times of the nicotine enantiomer pairs ranged from 7.64 and 8.34 min to 13.47 and 14.97 min, with the S(−) enantiomer eluting first. As expected, faster flow rates of 1.0 mL/min reduced retention times by approximately 1–2 min, with a slight decrease in the *R* values. The %RSD values for both resolution and retention times consistently remained below 2%. The detection limits for the enantiomers were approximately 5 μg/mL. The optimized method successfully detected one part in 100 for the minor R(+) enantiomer in the presence of the dominate S(−) enantiomer and adhered to all established QuEChERS method protocols.

## Graphical abstract

Because of the known pharmaceutical differences for the enantiomers of nicotine, precise analytical methods are required. Described herein is an optimized chiral high‐performance liquid chromatographic method for the quantitative and quantitative distribution of these enantiomers.

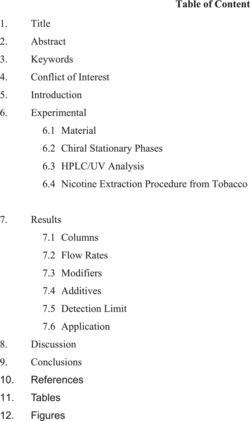

## 1 Introduction

Synthetic nicotine optical isomers are being produced in the laboratory and used in e‐cigarettes, with pure and mixtures of the L(−, S) and D(+, R) nicotine isomers being employed. Importantly, the pharmacological activities of the two nicotine enantiomers differ significantly [1], making it crucial to quantifying their distribution. As a result, the quantitative and qualitative assessments of nicotine isomer distribution, along with that of its related secondary alkaloids, have garnered considerable interest.

The United States Food and Drug Administration (FDA) has established regulations for the use of synthetic nicotine‐often referred to as tobacco‐free nicotine (TFN)—similar to those for tobacco‐derived nicotine (TDN) products. It is important to note that modern synthetic methods allow for the production of each nicotine enantiomer in pure form [1]. In parallel, the FDA has mandated that all combustible tobacco products must have lower nicotine content than those currently on the US market, with a maximum target level of 0.5 mg/g of tobacco. This mandate has driven the tobacco industry to develop new methodologies and agronomic practices to reduce the nicotine content in cured tobaccos (currently around 5 mg/g), or to employ *Nicotiana tabacum* cultivars that naturally contain lower levels of nicotine and its secondary alkaloids.

Historical data on the D/L (R/S) ratio of nicotine and its secondary alkaloids in these lower nicotine experimental tobaccos are lacking. Hence, robust and relatively rapid analytical methodologies for the qualitative and quantitative analysis of nicotine enantiomers and their secondary alkaloid counterparts are essential not only from a regulatory standpoint but also from an agronomic perspective.

To address these and other possible issues with nicotine, reports have appeared in the literature describing robust methods for the qualitative and quantitative analysis of nicotine enantiomers. The dominant and, arguably, the most consistently reported method for the assessments of nicotine enantiomer distributions has resided with applications of chiral high‐performance liquid chromatography (HPLC) and chiral supercritical fluid chromatography (SFC) [1, 2, 3, 4, 5, 6, 7, 8, 9, 10, 11, 12, 13, 14, 15]. Significant advances over the last few years have rendered these chiral methods meeting most of the criteria set forth in the QuEChERS method description, which is quick, easy, cheap, effective, rugged, and safe [6, 7].

As with any analytical method used to support compliance with federal regulations, having a complementary second method based on alternative chromatographic separation technology enhances data reliability and provides additional confirmation for regulatory compliance. To strengthen this documentation, a new chromatographic method has been developed, building on previous work with chiral liquid and SFC [8, 9, 10, 11, 12, 13, 14, 15]. This method reports both qualitative and quantitative data on the enantiomeric distribution of nicotine and clearly indicates its possible application to the analysis of enantiomers of related secondary alkaloids. Recent publications on chiral SFC have documented the qualitative and quantitative analysis of the nicotine and nornicotine enantiomers using this technique [12]. This new chiral SFC method meets all of the QuEChERS criteria.

The impact of modifiers and additives on the resolution and retention time of the nicotine enantiomers in Chiral SFC was recently reported [12]. The study explored the effects of different additives isopropylamine (IPAm), diethylamine (DEA), trimethylamine (TMA), and ammonium acetate (NH_4_OAc)), fortified into the modifier solution (ethanol and isopropanol, 0.2%). By varying the percentages of isopropanol and ethanol as modifiers, the retention times of the nicotine enantiomer pairs shifted from 5.23/5.66 min to 2.53/2.65 min. A relatively high ethanol modifier concentration (20%) let to a near‐complete loss of enantiomer resolution. In contrast, using a mobile phase containing 10% ethanol and 0.2% DEA, or 10% isopropanol with 0.2% DEA, resulted in a resolution factor (*R*) ranging from less than 1 to greater than 3. Based on these findings, it was fully anticipated that variations in the amount and type of modifiers and additives could significantly influence the resolution (*R*) and retention times of nicotine enantiomers under chiral HPLC conditions. In general terms, SFC has an advantage over traditional HPLC because the mobile phase composition is most often dominated by supercritical carbon dioxide, whereas the mobile phase in HPLC is most often dominated by organic solvents such as hexane, methanol, and acetonitrile that must be handled and disposed of properly. Alternatively, the hardware employed most often in HPLC applications is notably less expensive than that of SFC. Thus, both approaches have their distinct advantages and disadvantages. This report presents the results from systematic variation in selected modifiers and additives on the normal phase HPLC separation of nicotine enantiomers, with a focus on ensuring that the parameters meet and follow the QuEChERS criteria, the results of which can be directly compared with those obtained previously from SFC.

## 2 Experimental

### 2.1 Materials

ACS grade isopropyl amine (IPA), DEA, triethylamine (TEA), tributylamine (TBA), ethylenediamine (EDA), trifluoroacetic acid (TFA), and nicotine were obtained from Sigma‐Aldrich (St Louis, MO). Hexane, ethanol (EtOH), and isopropanol (IPOH) were HPLC grade and were obtained from Fisher Scientific (Pittsburgh, PA). All reagents were used as received.

### 2.2 Chiral Stationary Phases

Packed columns with CHIRALCEL OD with cellulose derivatives, CHIRALPAL IA, IC, IG‐3, IB N‐3, and AY‐3 with amylose derivatives (250 × 4.6 mm, dp = 3 μm), were obtained from Chiral Technologies, Inc. (West Chester, PA). All columns were conditioned as recommended by the manufacturer before use and tested with different modifiers and additives.

### 2.3 HPLC/UV Analysis

An Agilent 1200 Series HPLC equipped with a quaternary pump, variable wavelength detector (VWD‐UV), auto liquid sampler (ALS), and oven heater set to ambient was employed with all columns and modifiers. All analyses were performed at 254 nm. A nicotine standard, consisting of both the R and S enantiomers dissolved in DCM or hexane was used to optimize the chromatography conditions. Injection volume was set to 10 μL for all analyses. All separations were obtained under isocratic conditions with hexane as the main component of the mobile phase.

The column was conditioned following the manufacturer's recommendations.

### 2.4 Nicotine Extraction Procedure From Tobacco

A VLN brand cigarette was purchased from a local grocery store. The extraction protocol employed herein was that validated by CORESTA participants for the quantitative extraction of nicotine from tobacco matrices. The tobacco from a couple of cigarettes was emptied into a standard coffee grinder and ground for 10–15 s and then used without further modification. First, 0.5 g of the ground tobacco sample was mixed with 10 mL of water, followed by the addition of 5 mL of 8 N NaOH. The mixture was shaken for 1 h using a ThermoFisher Scientific shaker. The supernatant was then isolated by centrifugation at 4000 RPM for 4 min. Next, 10 mL of hexane was added to the extract and shaken for 1 h. The hexane layer was separated, and the extraction was repeated with an additional 10 mL of hexane. The combined hexane solutions were dried over sodium sulfate. Finally, the hexane extract was analyzed using an optimized HPLC method to determine the distribution of nicotine enantiomers.

## 3 Results

Building on the approach established in several earlier publications on HPLC and SFC nicotine enantiomer separations [1, 2, 3, 4, 5, 6, 7, 8, 9, 10, 11, 12], a systematic variation in the structure and concentration of modifier and additive was undertaken to develop a QuEChERS method for the chiral HPLC separation of the nicotine enantiomers. These variations included methodical changes to flow rate, modifiers type, additives type, and their respective concentrations. The modifiers tested included ethanol (EtOH), isopropanol (IPOH), and methanol (MeOH), whereas the additives included DEA, TEA, TBA, IPAm, EDA, and TFA.

### 3.1 Columns

Initially, six different chiral columns (IA, OD, IC, IG‐3, IB N‐3, and AY‐3) were screened using a 95/5% hexane/(IPA + 0.5% IPAm) mobile phase to determine which column provided the shortest retention time with the highest resolution. The preliminary results indicated that the IB N‐3 column offered the best resolution with nicotine enantiomer retention times under 15 min. In keeping with the QuEChERS method philosophy of making the protocol quick, therefore, all subsequent optimizations were performed using the IB N‐3 column.

### 3.2 Flow Rates

Varying the mobile phase flow rates between 1.0 and 0.8 mL/min resulted in changes to both the retention times and resolution (*R*) values of the nicotine enantiomer, as shown in Table 1 (EtOH = ethanol, IPOH = isopropanol), with the S(−) nicotine enantiomer eluting from the column first. The influences of both column type and mobile phase flow rates on *R* values and retention times were shown to be relatively insignificant when compared with the influences on these values when changes in mobile phase composition were made, *vide infra*.

**TABLE 1:** Variation in nicotine enantiomers retention times (minutes/minutes) and resolution (*R*), as functions of modifier type, mobile phase flow rate.^a^ See Section 2 for chromatographic conditions.

Modifier	% Modifier	Flow rate, mL/min	Retention time, min	Resolution, *R*
EtOH	3.5	1.0	6.88/7.36	3.8
EtOH	3.5	0.8	8.27/9.42	4.3
EtOH	5.0	1.0	6.12/6.50	3.4
EtOH	5.0	0.8	7.69/8.17	3.6
IPOH	3.5	1.0	8.32/9.05	4.4
IPOH	3.5	0.8	10.35/11.23	4.4
IPOH	5.0	1.0	7.26/7.82	3.7
IPOH	5.0	0.8	9.14/9.85	3.9

^a^ Constant additive, diethylamine (DEA), at 0.2%.

Increasing the flow rate reduced the retention time of the nicotine enantiomers, along with a corresponding decrease in resolution (*R*). Additionally, using ethanol (EtOH) instead of isopropanol (IPOH) at the same flow rates led to differences in both the retention times and resolution (*R*) of the nicotine enantiomers.

### 3.3 Modifiers

The use of different alcohol as a modifier in the mobile phase, along with varying amine additives, resulted in noticeable difference in resolution (*R*) values and retention times, as shown in Tables 1 and 2, respectively.

**TABLE 2:** Impact of alcohol modifiers and additives on the retention time and resolution of nicotine enantiomers. See Section 2 for the chromatography conditions.

Modifier	% Modifier	Additive	% Amine	Res.	% RSD	Ret. time, min	% RSD
Isopropanol	2	Diethylamine	0.1	Long tR w/tailing	ND^a^	Long tR w/tailing	ND^a^
Isopropanol	2	Diethylamine	0.5	Long tR w/tailing	ND	Long tR w/tailing	ND
Isopropanol	5	Diethylamine	0.1	3.1	5.8	10.46/9.60	0.2
Isopropanol	5	Diethylamine	0.5	3.9	0.9	10.05/9.25	3.3
Isopropanol	3.5	Diethylamine	0.2	4.4	2.7	11.23/10.35	0.6
Isopropanol	5	Diethylamine	0.2	3.9	6.1	9.85/9.14	1.3
Isopropanol	2	Triethylamine	0.1	Long tR w/tailing	ND	Long tR w/tailing	ND
Isopropanol	2	Triethylamine	0.5	4.6	2.6	14.97/13.47	0.5
Isopropanol	3.5	Triethylamine	0.2	4.0	3.1	11.92/10.90	0.6
Isopropanol	5	Triethylamine	0.1	3.2	2	10.70/9.85	0.5
Isopropanol	5	Triethylamine	0.5	3.8	1.7	10.39/9.54	0.4
Isopropanol	2	Isopropylamine	0.1	Long tR w/tailing	ND	Long tR w/tailing	ND
Isopropanol	2	Isopropylamine	0.5	Long tR w/tailing	ND	Long tR w/tailing	ND
Isopropanol	3.5	Isopropylamine	0.2	3.9	2.8	11.82/10.76	0.6
Isopropanol	5	Isopropylamine	0.1	3.8	3.2	10.17/9.31 (tailing)	0.6
Isopropanol	5	Isopropylamine	0.5	4.2	2.9	10.24/9.39	0.01
Ethanol	3.5	Diethylamine	0.2	4.3	6.5	9.11/8.36	0.7
Ethanol	5	Diethylamine	0.2	3.6	0.1	8.18/7.69	0.01
Ethanol	5	Diethylamine	0.1	4.4	2.0	8.46/7.76	0.3
Ethanol	2	Diethylamine	0.1	7.5	0.7	12.05/10.61	0.4
Ethanol	2	Diethylamine	0.5	7.6	2.4	11.27/9.99	0.3
Ethanol	5	Diethylamine	0.5	4.7	0.8	8.34/7.64	0.3
Ethanol	2	Triethylamine	0.1	5.2	1.6	11.34/10.40	0.4
Ethanol	2	Triethylamine	0.5	7. 6	1.1	11.88/10.41	2.0
Ethanol	3.5	Triethylamine	0.2	4.8	2.1	9.11/8.36	0.7
Ethanol	5	Triethylamine	0.1	4.9	0.4	8.27/7.56	0.3
Ethanol	5	Triethylamine	0.5	4.8	0.4	8.42/7.71	0.1
Ethanol	2	Isopropylamine	0.1	5.6	2.1	9.77/8.83	1.8
Ethanol	2	Isopropylamine	0.5	5.7	0.9	10.78/9.75	0.2
Ethanol	3.5	Isopropylamine	0.2	4.4	5.9	8.70/7.95	0.6
Ethanol	5	Isopropylamine	0.5	4.4	3.4	8.18/7.47	0.5
Ethanol	5	No additive	0	3.9	0.7	8.35/7.56	ND^a^
Ethanol/MeOH^b^	3.5	Diethylamine	0.2	2.9	ND	9.58/9.03	7.8
Ethanol/MeOH^b^	2	Diethylamine	0.5	6.5	ND	10.77/9.80	6.5
Ethanol	5.0	Tributylamine	0.1	3.9	ND	8.72/7.98	0.5
Ethanol	5.0	Ethylenediamine	0.1	4.4	ND	7.94/7.29	2.0
Ethanol	2	Diethylamine/TFA	0.5 + 0.1	7.6	ND	10.85/9.64	ND^a^ ^,^ ^b^
Ethanol	2	Diethylamine/TFA	0.5 + 0.2	7.5	ND	10.89/9.69	ND^a^ ^,^ ^b^
Ethanol	2	Diethylamine/TFA	0.5 + 0.5	Not eluted	ND	Not eluted	ND

^a^ Not determined.

^b^ 50/50% volume.

Varying the percentage of alcohol in the mobile phase modifier from 2% to 5%, while keeping amine additive levels constant, resulted in changes in nicotine enantiomer resolution (*R*), with values ranging from 4.9 to 7.57 for ethanol and DEA. Additionally, altering the structure of the mobile phase modifier—using ethanol, isopropanol, or an ethanol/methanol mixture—also affected the *R* values, yielding 4.7 for ethanol compared with 3.11 for isopropanol at 0.1% DEA.

When ethanol was used as the modifier with a constant amine additive level of 0.1%, the *R* values for nicotine enantiomers showed little variation (approximately 4.7) across different amine additives, except for TBA, which resulted in an *R* value of 3.9. When only 5% ethanol was employed as a modifier without any additive, the *R* value for the nicotine enantiomers was 3.9 (%RSD = 0.5), with corresponding retention times of 8.70 and 7.99 min.

Using isopropanol as a mobile phase modifier occasionally led to undesirable chromatographic behavior, such as peak tailing, which was not observed with either ethanol or the ethanol/methanol mixture. Furthermore, the *R* values for 2% ethanol were consistently higher than those obtained with 3.5% or 5% ethanol, regardless of the type or percentage of amine additive used.

Varying the percentage of alcohol mobile phase modifier from 5% to 2%, while keeping the amine additive levels constant, resulted in changes to the retention times of nicotine enantiomers. For example, the retention times shifted from 7.75/8.45 min to 10.61/12.05 min when using ethanol and DEA. Additionally, changing the structure of the mobile phase modifier—using ethanol, isopropanol, or ethanol/methanol mixture—also affected the retention times. Specifically, the retention times were 8.78/9.42 min for 3.5% ethanol, 10.35/11.23 min for isopropanol, and 9.03/9.58 min for the ethanol/methanol mixture, at 0.2% DEA.

### 3.4 Additives

With ethanol held constant at 5% and amine additive levels at 0.1%, there was little change in the retention times of nicotine enantiomers when five different amine additives were employed, resulting in average retention times of 7.65 and 8.35 min, with a range of ±0.3 min. When only 5% ethanol was used as a modifier without any additive, the retention times were 7.99 and 8.70 min.

Using isopropanol as a mobile phase modifier occasionally led to undesirable chromatographic behavior, characterized by relatively long retention times when DEA and IPAm were used as additives. This behavior was not observed with ethanol or the ethanol/methanol mixture as mobile phase modifiers. Systematic stepwise changes in both modifiers and additives resulted in corresponding stepwise changes in nicotine enantiomer retention times. Notably, regardless of the additive type or percentage, the retention times for nicotine enantiomers at 2% ethanol were consistently higher than those obtained with 3.5% or 5% ethanol. Additionally, the level of additives was shown to influence the peak shapes of nicotine enantiomers across a wide range of modifier percentages.

In a number of literature citations, TFA has been employed along with DEA as a second coadditive and ethanol as a modifier in the chiral HPLC separation of nicotine enantiomers [1, 4, 13]. The influence of TFA on the separations investigated herein was studied by adding selected amounts of TFA (0.1%, 0.2%, and 0.5%) to a mobile phase consisting of 98% hexane, 2% ethanol, and 0.5% DEA. The nicotine enantiomer *R* values for these three mobile phases were, respectively, 7.6, 7.5, and no observed enantiomer elution.

### 3.5 Detection Limit

Authentic gravimetric standards of nicotine enantiomers were prepared at 5 and 10 ng/μL, and optimized chromatographic conditions were employed for their separations with UV detection at 254 nm. The results indicate that a reasonable nicotine enantiomers detection limit of approximately 10‐ng mass injected can be attained with an accompanying signal‐to‐noise ratio of at least 3/1.

### 3.6 Application

The presence and distribution of nicotine enantiomers were determined with the chiral HPLC analysis of tobacco taken from a commercially available low alkaloid cigarette using the optimum conditions obtained in this study (Chiral column IB N‐3, flow of 0.8 mL/min of 98/2% hexane + ethanol (0.5% DEA [v/v]). The distribution of nicotine enantiomers was very similar to that appearing in the literature.

## 4 Discussion

**FIGURE 1:**
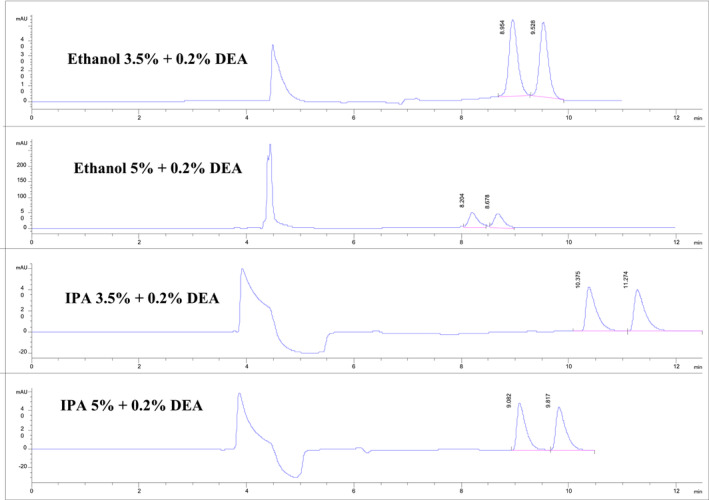
Chiral HPLC/UV/VIS separations of nicotine enantiomers as a function of mobile phase modifier and constant additive percentage. See Section 2 for separation details.

**FIGURE 2:**
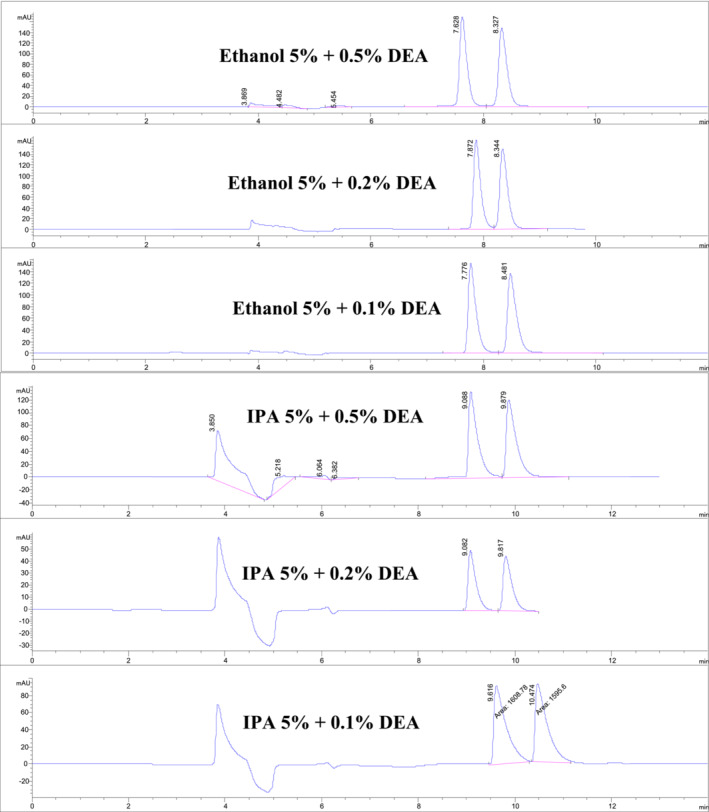
Chiral HPLC/UV/VIS separations of nicotine enantiomers as a function of mobile phase additives and constant modifier percentage.

**FIGURE 3:**
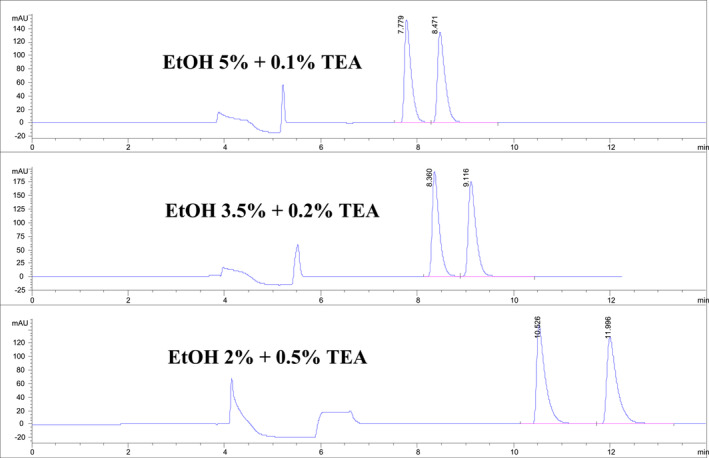
Chiral HPLC/UV/VIS separations of nicotine enantiomers as a function of systematic changes in alcohol modifier and additive levels. Flow 0.8 mL/min. See Section 2 for chromatography conditions.

Systematic variations in normal phase HPLC mobile phase flow rates (between 1.0 and 0.8 mL/min) and mobile phase modifier levels produced significant changes in nicotine enantiomers retention times and with no accompanying shifts in nicotine enantiomer *R* values, as shown in Tables 1 and 2 and Figures 1, 2, and 3. For example, increasing the 3.5% ethanol mobile phase flow rate from 0.8 to 1.0 mL/min precipitated a marked reduction in retention times for the nicotine enantiomers, 9.42/8.77 to 7.38/6.88 min, respectively. Similar shifts in nicotine enantiomer retention times were observed when isopropanol was used as a mobile phase modifier. However, no significant changes in nicotine enantiomer *R* values were observed with changes in flow rates or mobile phase modifier. Somewhat surprisingly, the use of isopropanol instead of ethanol did precipitate in a number of instances undesirable enantiomer peak shapes with pronounced tailing. Thus, in these normal phase HPLC separations with hexane as the dominant mobile phase component, ethanol possesses a more desirable chromatographic over a larger range of concentrations than did isopropanol.

In the vast number of cases, the nicotine enantiomers were well separated, with more than baseline resolution, with well‐shaped peaks, and retention times under 15 min for both modifiers and additives, meeting several critical parameters of the QuEChERS protocol. Systematic changes in both alcohol modifiers and amine additives to the mobile phase produced orderly shifts in retention times and shifting *R* values (Figure 3).

The chiral HPLC chromatograms revealed stepwise increases in nicotine enantiomer retention times and a significant increase in nicotine enantiomer *R* value between 3.5% and 2% ethanol. This finding illustrates the extremely critical impact of modifier percentage on the nicotine enantiomer *R* values and retention times. Judicious selection of the values for modifier and additive type and levels can thus lead to meaningful shifts in both retention times and enantiomer *R* values improving the performance of the analytical method by meeting a number of the QuEChERS protocol requirements.

**FIGURE 4:**
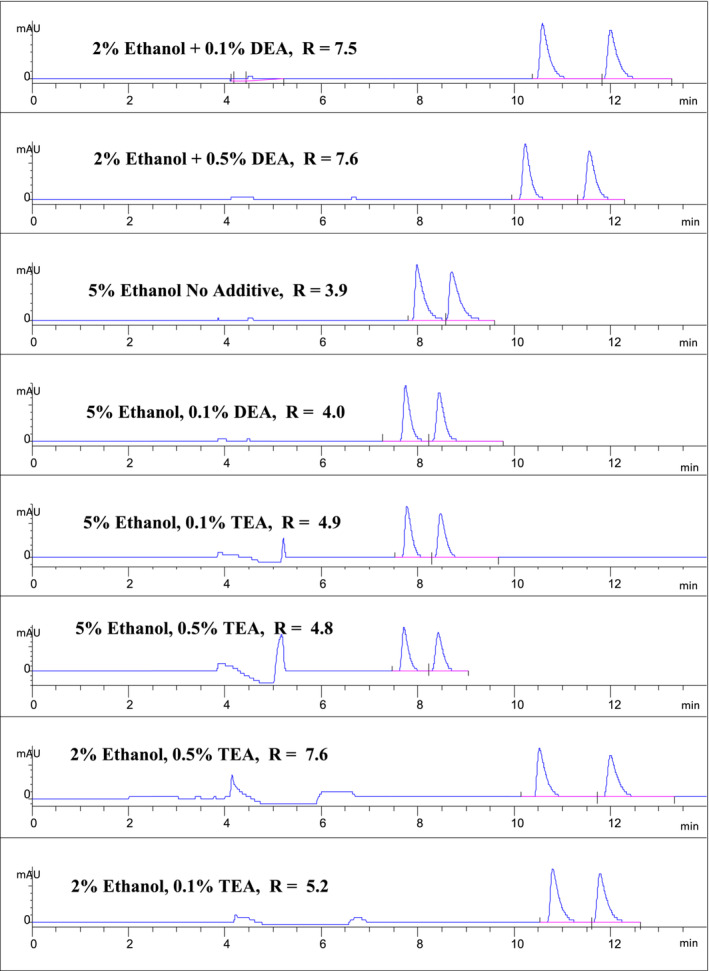
Influences of HPLC mobile phase additives on the nicotine enantiomer peak shape at selected modifier and additive levels. See Section 2 for the extraction and analysis method.

**FIGURE 5:**
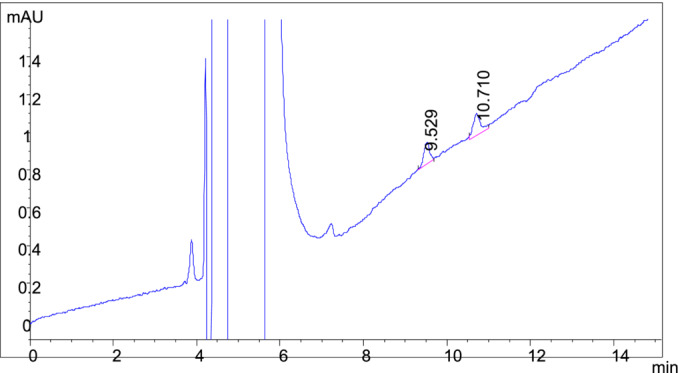
Chiral HPLC chromatogram of nicotine enantiomers at a nicotine concentration of 5 ng/μL, 2 μL injected. See Section 2 for the extraction and analysis method.

A convenient manner in which to view the interplaying performances of both the mobile phase modifiers and mobile phase additives is in the form of data presented in a 3 × 3 matrix (Figures 4 and 5).

The 3 × 3 matrix data within Table 3 clearly reveals the notable nonstepwise/abrupt increase in nicotine enantiomer *R* value when the percentage ethanol modifier drops to the low value of 2% and the amine additive reaches 0.5%. The 3 × 3 matrix data within Table 4 clearly reveals that the nicotine enantiomer retention times significantly increase systematically with the decrease in ethanol mobile phase modifier increase while the influence of the changes in additive percentage is not as clear. Such additional 3 × 3 matrices can be developed from the data.

**TABLE 3:** A 3 × 3 matrix presentation of the influences of ethanol modifier and triethylamine additive percentages on the *R* values of nicotine enantiomers.

Ethanol	Triethylamine additive%		
Alcohol modifier %	0.1	0.2	0.5
**2**	**5.2**		**7.6**
**3.5**		**4.8**	
**5**	**4.6**		**4.8**

**TABLE 4:** A 3 × 3 matrix presentation of the influences of ethanol modifier and isopropylamine additive percentages on the retention times values of nicotine enantiomers.

Ethanol	Isopropyl amine additive%		
Alcohol modifier %	0.1	0.2	0.5
**2**	**8.83/9.77**		**9.75/10.78**
**3.5**		**7.95/8.7**	
**5**	**7.69/8.38**		**7.47/8.18**

Among the protocol requirements, a method to meet QuEChERS requirements is effective and rugged. These criteria can be met when the method of analytical data supports analyte specificity/accuracy and precision. From the data contained herein, the presence of baseline enantiomer resolution coupled with detection at 254 nm provides strong supporting evidence for specific analyte detection. %RSD values for both analyte retention times and analyte enantiomer *R* values at less than 3% support the rugged character of the approach.

A critical and important aspect of successful chromatographic separations is the nature of the peak shapes of the analytes of interest. The influence of additive percentage on the shape of the nicotine enantiomer peaks is clearly evident (Figure 4).

Some significant observations on peak shape as well as other chromatographic characteristics can be garnered from Figure 4. Noticeable and meaningful positive improvements in nicotine enantiomer peak shapes occurred when the DEA levels changed with a 2% ethanol modifier with no meaningful changes in nicotine enantiomer retention times. At 5% ethanol and 0.1% DEA, 0.1% TMA, and 0.5% triethylamine, improvement in peak shapes and a slight shift in retention times were observed for the nicotine enantiomers. Increasing levels of triethylamine at a constant 2% ethanol concentration improved the nicotine enantiomer peak shape with little impact on their retention times. These findings with ethanol as a modifier coupled with those observed when isopropanol was employed as a modifier clearly revealed the two alcohols; ethanol provided superior overall performance characteristics.

The influence of TFA on the nicotine enantiomer *R* value was investigated by adding sequential amounts of TFA (0.1%, 0.2%, and 0.5%) to a mobile phase consisting of 98% hexane, 2% ethanol, and 0.5% DEA (Table 2). For the 0.1% and 0.2% cases, no measurable changes in nicotine enantiomer *R* values, 7.6, and 7.5, respectively, were noted when compared with the *R* value for the same mobile phase with no TFA added of 7.57. Interestingly, and somewhat unexpectedly, when the TFA concentration in the mobile phase was increased to 0.5%, no nicotine enantiomer elution was observed. This behavior is likely due to the fact that compounds should ideally be in a neutral state when analyzed on polysaccharide chiral stationary phases, such as employed herein. Introducing a counter ion additive, which charges the compound, can lead to strong ionic interactions with the selectors, typically resulting in prolonged retention or significant tailing. In this case, it appears that as more TFA is added, the nicotine becomes protonated and remains bound to the column, preventing elution.

Overall, this result highlights the importance of making incremental adjustments to additive and modifier concentrations when assessing their effects on enantiomeric resolution (*R* values) and retention times, particularly when optimizing methods to comply with QuEChERS protocols. Obviously, there is little to no reason for creating mobile phase compositions that result in enantiomeric *R* values greater than one. However, it is critically important to fully understand how the mobile phase compositions influence *R* values and retention times, the main foci of this manuscript, and how subtle changes in mobile phase compositions can actually result in loss of enantiomer elution.

An additional important criterion for a QuEChERS method is the ability of the approach to detect the analyte(s) of interest at concentrations closely linked to levels of the analyte(s) appearing in samples of interest. Figure 5 presents the chiral HPLC chromatogram of nicotine enantiomers at a nicotine concentration of 5 ng/μL, 2 μL injected. Both enantiomers are detected at signal‐to‐noise levels greater than 3/1, and the presence of the minor nicotine enantiomer is detected in the presence of the major nicotine enantiomer at a ratio of approximately 1/100.

**FIGURE 6:**
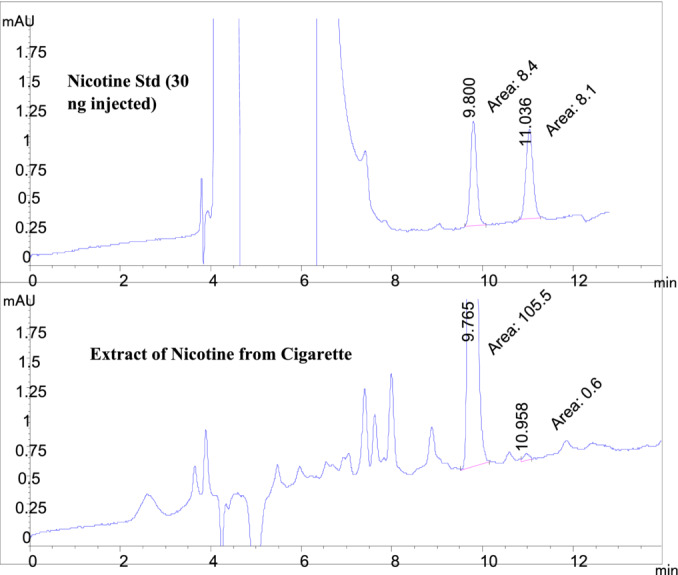
Chiral HPLC analysis of nicotine enantiomers in a commercial cigarette extract. See Section 2 for the extraction and analysis method.

The US Food and Drug Administration published an Advanced Notice of Proposed Rulemaking (ANPRM) in March of 2018 titled “Tobacco Product Standard of Nicotine Level of Certain Tobacco Products.” The ANPRM addressed reducing nicotine levels in tobacco fillers used for cigarette manufacture by 95% to 98% [16]. A standard cigarette filler contains 15–25 mg of nicotine per gram of tobacco. In a similar vein, the World Health Organization recommends a 35‐fold reduction, which corresponds to 0.4 mg/g or 0.04% of nicotine [17]. Application of this nicotine enantiomer analysis protocol to the analysis of the tobacco contained within a commercially available low‐alkaloid tobacco cigarette revealed that the method was appropriate for the qualitative and quantitative analysis of the nicotine enantiomeric distribution of the sample (Figure 6). The distribution of nicotine enantiomers, S (−), at ~99.4% and R(+) at ~0.6%, is well within the ranges found for selected tobacco cultivars, cigarette tobacco fillers, and smokeless tobacco [18]. The protocol was able to detect the presence of both isomers with acceptable signal‐to‐noise ratios.

## 5 Conclusions

The main focus of these data has been to present conclusive documentation of the influences of subtle changes in mobile phase modifiers and additives on the chiral HPLC chromatography with variable wavelength UV detection for the determination of the enantiomer distributions of nicotine and how knowledge of these subtle systematic changes in these parameters critically influence the creation of an effective overall enantiomeric separation. Presumably, the improvements in peak shapes could be attributed to the basic character of the amines that resulted in their occupation of active sites that otherwise might have interacted with the nicotine enantiomers. In a similar fashion, the addition of alcohol modifiers presumably altered the solubility of the nicotine enantiomers, precipitating changes in their retention times. Optimizing the levels of mobile phase modifiers and additives to produce a QuEChERS method was achieved with the use of an inexpensive mobile phase solvent, alcohol modifiers, and amine additives at relatively low concentrations. The method is both accurate and precise with baseline enantiomer separations in less than 12 min and detection limits in the range of 5 ng/μL coupled with %RSD values consistently less than 3% for both nicotine enantiomer retention times and *R* values. The influences of column type and mobile phase flow rates on enantiomer separations were shown to be minor when compared with the influences of *R* values and retention times observed due to changes in mobile phase compositions. When compared with the results obtained previously employing an SFC‐based separation, both methods were found to meet QuEChERS criteria illustrating the advantages and disadvantages of both approaches. Of note is a comparison between the HPLC and SFC enantiomeric retention times and enantiomeric resolution with comparable values obtained by gas chromatography (GC) [19]. Although the HPLC and SFC protocols referenced and discussed herein have enantiomer retention times equal to or less than 10 min, and *R* values much greater than one, the GC values are retention times greater than 150 min with much less desirable *R* values. Successful application of this chiral HPLC protocol using the optimum conditions obtained in this study (Chiral column IB N‐3, flow of 0.8 mL/min of 98/2% hexane + ethanol (0.5% DEA [v/v]) has been demonstrated through the analysis of the nicotine enantiomer distribution within the tobacco filler contained in a commercially available low alkaloid cigarette.

## Data Availability

All data are available for review upon request.
